# Epitope Specific Antibodies and T Cell Receptors in the Immune Epitope Database

**DOI:** 10.3389/fimmu.2018.02688

**Published:** 2018-11-20

**Authors:** Swapnil Mahajan, Randi Vita, Deborah Shackelford, Jerome Lane, Veronique Schulten, Laura Zarebski, Martin Closter Jespersen, Paolo Marcatili, Morten Nielsen, Alessandro Sette, Bjoern Peters

**Affiliations:** ^1^Center for Infectious Disease, La Jolla Institute for Allergy and Immunology, La Jolla, CA, United States; ^2^Department of Bio and Health Informatics, Technical University of Denmark, Kongens Lyngby, Denmark; ^3^Instituto de Investigaciones Biotecnológicas, Universidad Nacional de San Martín, Buenos Aires, Argentina; ^4^University of California San Diego, La Jolla, CA, United States

**Keywords:** IEDB, epitope, antibody, TCR, BCR, CDR, repertoire sequencing, AIRR

## Abstract

The Immune Epitope Database (IEDB) is a free public resource which catalogs experiments characterizing immune epitopes. To accommodate data from next generation repertoire sequencing experiments, we recently updated how we capture and query epitope specific antibodies and T cell receptors. Specifically, we are now storing partial receptor sequences sufficient to determine CDRs and VDJ gene usage which are commonly identified by repertoire sequencing. For previously captured full length receptor sequencing data, we have calculated the corresponding CDR sequences and gene usage information using IMGT numbering and VDJ gene nomenclature format. To integrate information from receptors defined at different levels of resolution, we grouped receptors based on their host species, receptor type and CDR3 sequence. As of August 2018, we have cataloged sequence information for more than 22,510 receptors in 18,292 receptor groups, shown to bind to more than 2,241 distinct epitopes. These data are accessible as full exports and through a new dedicated query interface. The later combines the new ability to search by receptor characteristics with previously existing capability to search by epitope characteristics such as the infectious agent the epitope is derived from, or the kind of immune response involved in its recognition. We expect that this comprehensive capture of epitope specific immune receptor information will provide new insights into receptor-epitope interactions, and facilitate the development of novel tools that help in the analysis of receptor repertoire data.

## Introduction

The adaptive immune system in vertebrates has evolved to recognize and combat an ever changing repertoire of pathogenic organisms such as viruses, bacteria, and parasites. The ability to recognize this plethora of attackers is vastly due to B and T lymphocytes which express a highly diverse repertoire of antigen receptors. Both B and T cell receptors are generated through a stochastic process in which segments from several genes are re-arranged (1). B cell receptors (BCRs) or antibodies (secreted BCRs) are typically heterodimers of two different proteins, a heavy and a light chain, while T cell receptors (TCRs) are made up of α and β or γ and δ chains. Chromosomes encoding the heavy and β chains proteins in every B- and T cells, respectively, have DNA modules composed of variable (V), diversity (D), joining (J), and constant (C) genes. On the other hand, light and α chains are encoded by modules of V, J, and C genes. For example, the IMGT database (2) reports 68 V, 2 D, 14 J, and 2 C genes in the human TCR locus of the β chain and 54 V, 61 J, and 1 C genes in the complementary α chain locus.

The recombination process rearranges one each of these possible V, D, and J gene segments to be adjacent to each other. B and T cells with productive rearrangements of the two chains express BCRs and TCRs on their surface, respectively. The protein domain encoded by V(D)J recombination in heavy and light chains is known as the variable domain. This combinatorial rearrangement process is the key to receptor diversity. Receptor diversity is further amplified by insertions and deletions at the junctions between the various gene segments (3). While TCRs are stable after this initial V(D)J re-arrangement, BCRs can further mutate due to somatic hypermutations and affinity maturation, resulting in even higher BCR diversity which is associated with high affinity with their cognate antigen (4). These processes ultimately supply the host with a broad array of BCR and TCR receptors capable of binding to immune epitopes that allow the immune system to distinguish self from non-self.

**Figure 1:**
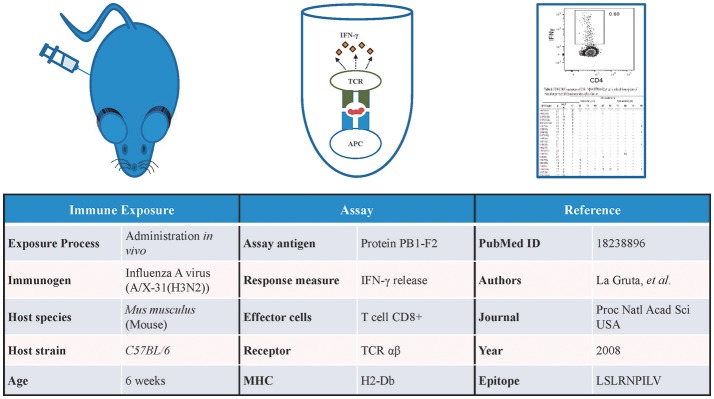
Information captured in the IEDB. Detailed information related to the immune exposure of the host, type of assay used to test the immune response, and the reference of the data is captured in the IEDB. Data shown in this figure is from IEDB Assay ID: 1479091.

The Immune Epitope Database (IEDB) contains data gathered by manual curation of the scientific literature and through direct submissions of experimentally identified B- and T-cell epitopes and MHC ligands (5). As of August 2018, the IEDB has over 462,000 epitopes from over 19,500 manually curated references and direct submissions. In addition to capturing the identity of these epitopes, the IEDB also captures a vast array of information on the host organism in which the epitope is recognized, immune exposures of the host that led to the epitope recognition, the type of immune response targeting the epitope, and the epitope specific TCRs or BCRs/antibodies (Figure 1).

Originally, BCR and TCR sequence information was only curated in the IEDB if a formal sequence record was available in GenBank or UniProt. This was nearly exclusively the case for 3D structures of receptor-epitope complexes, as immune receptor sequencing was expensive and labor intensive. However, with the advent of next generation receptor sequencing experiments, also known as Rep-Seq (6), epitope specific BCR and TCR sequences are increasingly becoming available. The sequence data from such experiments is typically limited to one of the two receptor chains, and often targets the highly variable CDR3 (Complementarity Determining Region 3). Capturing these data appropriately and making it compatible with the existing full length receptor sequence data in the IEDB required modifying the IEDB curation approach and database design, as well as the query and reporting interfaces. These changes are described in the present article.

## Changes in the IEDB database structure and curation process for immune receptors

### Extension of information captured on immune receptors

In the past, IEDB receptor data was captured as part of the B- and T-cell assay tables, and included the receptor names (e.g., OT-2), types (e.g., α/β), isotypes (e.g., IgG4), immunoglobulin (Ig) domains (e.g., Fab, Fv, Whole antibody) and links to their sequence records (e.g., UniProt or NCBI accessions) for each of the chains (Table 1). As pointed out, above, next generation immune receptor sequencing experiments often provide partial receptor sequences. To store this information, we added fields to capture CDR1, CDR2, and CDR3 amino acid sequence information, as well as VDJ gene usage (Table 1). We used the IMGT definition for CDRs (7), and followed the WHO-IUIS nomenclature for VDJ genes (8). As sequencing experiments often target nucleotide sequences, a field to store them was also added to the assay table (See Table 1).

**Table 1:** Data structure and grouping of captured receptor information.

**Data fields**	**Assay receptor**		**Distinct receptor**	**Receptor group**
Receptor name	PMEL17			
Source organism	Homo sapiens		Homo sapiens	Homo sapiens
Sequence identifier	Chain1: NCBI:5EU6_D Chain2: NCBI:5EU6_E			
Protein sequence	Chain1: MKQEVTQIPAALS… Chain2: GAGVSQTPSNKVT…			
Nucleotide sequence	–			
	**Curated**	**Calculated**		
V gene	Chain1: TCRAV21 Chain2: TCRBV7-3	Chain1: TRAV21^*^01 Chain2: TRBV7-3^*^01	Chain1: TRAV21^*^01 Chain2: TRBV7-3^*^01	
D gene	–	–	–	
J gene	–	Chain1: TRAJ53^*^01 Chain2: TRBJ2-3^*^01	Chain1: TRAJ53^*^01 Chain2: TRBJ2-3^*^01	
Receptor type	αβ	αβ	αβ	αβ
Chain type	Chain1: α Chain2: β	Chain1: α Chain2: β	Chain1: α Chain2: β	Chain1: α Chain2: β
Variable domain sequence	–	Chain1:KQEVTQIPA… Chain2:AGVSQTPSN…	Chain1:KQEVTQIPA… Chain2:AGVSQTPSN…	
CDR1 sequence	Chain1:DSAIYN Chain2:SGHTA	Chain1:DSAIYN Chain2:SGHTA	Chain1:DSAIYN Chain2:SGHTA	
CDR1 positions	–	Chain1: 28-33 Chain2: 27-31		
CDR2 sequence	Chain1:IQSSQRE Chain2:FQGTGA	Chain1:IQSSQRE Chain2:FQGTGA	Chain1:IQSSQRE Chain2:FQGTGA	
CDR2 positions	–	Chain1: 51-57 Chain2: 49-54		
CDR3 sequence	Chain1: AVLSSGGSNYKLTF Chain2: ASSFIGGTDTQYF	Chain1: AVLSSGGSNYKLT Chain2: ASSFIGGTDTQY	Chain1: AVLSSGGSNYKLT Chain2: ASSFIGGTDTQY	Chain1: AVLSSGGSNYKLT Chain2: ASSFIGGTDTQY
CDR3 positions	–	Chain 1: 92-104 Chain 2: 93-104		

*Receptor data captured from publications is shown in ‘assay receptor’ column (IEDB assay ID: 2723539). The values in distinct receptor column were used for creating distinct receptor entries by combining receptors from different assays. If variable domain sequence was not available then CDR 1, 2 and 3 sequences were used to create distinct receptors. Similarly, the values in receptor group column are used for clustering similar distinct receptors in a group*.

**Figure 2:**
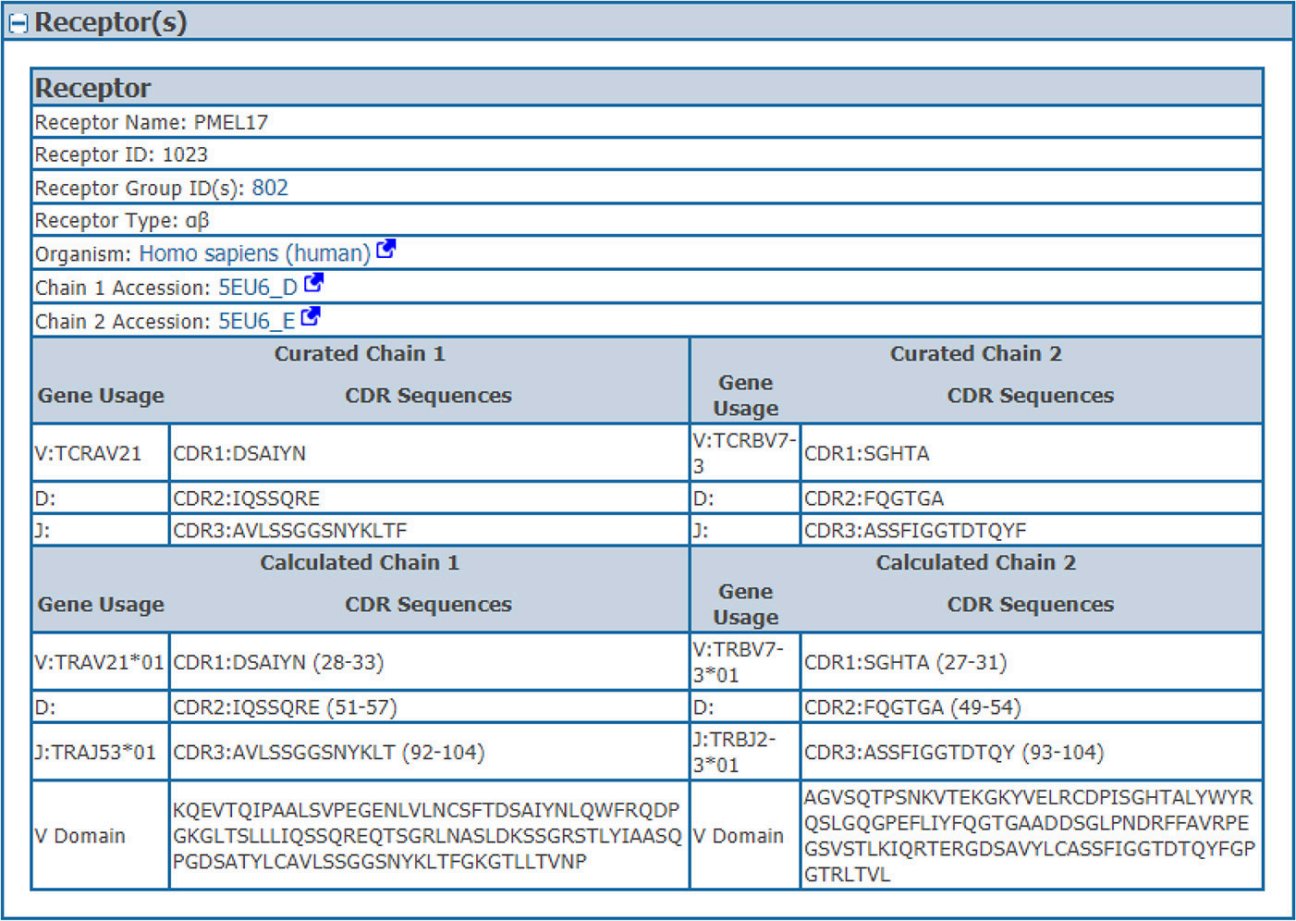
Assay receptors. The curated and calculated assay receptor information is displayed side by side on the assay details pages in the IEDB. Data shown in this figure is from the IEDB Assay ID: 2723539.

We wanted to capture the same information on CDRs and gene usage for receptor data for which full length protein sequences were previously curated. Thus, we identified CDRs, their position in the full length sequence, variable domain sequences and VDJ gene usage from full chain protein sequences based upon the IMGT numbering scheme (7) using ANARCI software v1.1 (9). This “calculated” information was stored in the assay table side by side with the “curated” information provided by the author if both are available (Table 1). The calculated and curated receptor information is displayed on the assay details pages in the IEDB (Figure 2).

### Distinct receptor identifiers

As we do for epitopes and assays, we wanted to assign numeric IEDB identifiers to receptors that serve as a stable reference, and group together all information available for a specific receptor studied. As an epitope database, the IEDB considers two immune receptors to be distinct if they have different specificities. For example, addition of a histidine tag to an antibody is not expected to significantly change its specificity, so we would want data from an antibody with and without such a tag to be grouped together, and want to assign it the same identifier to be able to interlink such reports. Similarly, differences in the nucleotide sequences of TCRs that encode for the same amino acid variable domain are not expected to result in different specificities. Based on these considerations, we identified the subset of information in Table 1 that is clearly linked to receptor specificity, namely the species of the host organism making the receptor, the receptor type, and the sequence of the variable domain/s. If the full length variable domain sequence is not available, all the available CDR sequences are considered. For several values, such as CDR3 regions, an assay may have both curated data (which reflects what the author stated to be the CDR3), and calculated data (which is based on automated analysis of the full length sequence). If both curated and calculated data are available and they are in conflict, we prioritize the calculated information, as it is easier for us to guarantee that it follows the IMGT numbering scheme. Overall, the rows in “distinct receptor” column of Table 1 identify the subset of properties that are used to identify distinct receptor entries, and which are linked to all assay entries that have receptors that match these fields.

### Receptor groups

While the definition of distinct receptors interlinks records for which the same receptor sequence information is given, it keeps records separate for which information is provided at different levels of granularity. For example, receptors for which only the TCR-beta chain is sequenced will be separated from receptors that have both the TCR-alpha and TCR-beta sequence available. Given that the CDR3 region of immune receptors is the most variable and is typically responsible for most contacts of the receptor with the epitope recognized, we decided to provide groups of receptor data that share the same CDR3 sequence.

**Figure 3:**
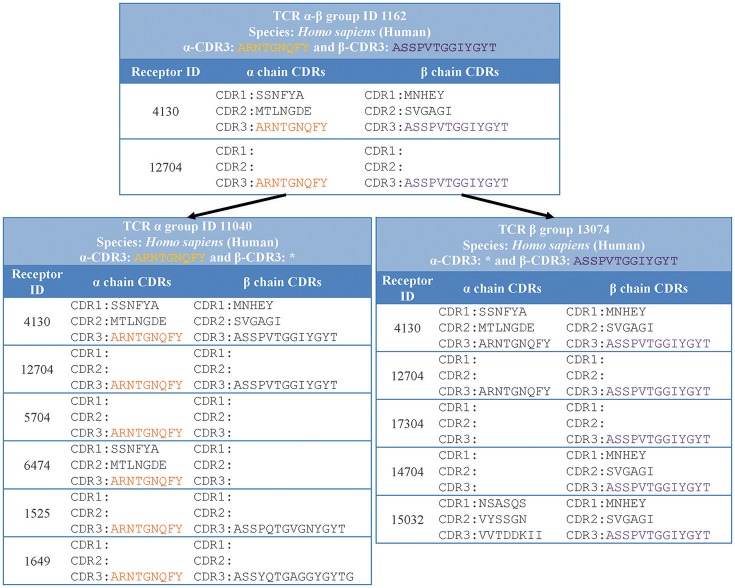
Receptor groups. Receptors are grouped based on their type, CDR3 sequence/s and host organism. Next generation repertoire sequencing experiments can report only a single chain CDR3 sequence for a receptor. Therefore, we group receptors hierarchically in groups with identical single chain CDR3 sequences (receptor group ID: 11040) which are divided in receptor groups based on CDR3 sequences from the other chain (receptor group ID: 1162 and 1525).

Specifically, we grouped together distinct receptors that had the same host species, receptor type, and CDR3 sequence/s (shown in “receptor group” column of (Table 1). This classification is hierarchical, so that the receptor group sharing the same TCR-α CDR3 sequence, can be subdivided into multiple receptor groups based on their TCR-β CDR3 sequence. Figure 3 illustrates how different distinct receptors are assigned to receptor groups. All the curated receptors were grouped into 18,292 receptor groups using above mentioned criteria.

### Receptor types: special cases

While the majority of vertebrates produce heterodimeric antibodies with heavy and light chains, camelids (camels, llamas and alpacas) produce naturally occurring heavy chain only antibodies devoid of light chains (HCAbs) (10). Similarly, sharks and other cartilaginous fish produce IgNARs (Immunoglobulin New Antigen Receptors) which are homodimeric heavy chain only antibodies (11). These observations have led to the development of engineered antibodies with a single heavy chain variable domain, known as V_H_H or nanobodies. Nanobodies and other types of antibody and TCR constructs, such as single chain antibodies (scFv), single chain TCRs (TscFv), single domain antibodies (sdAbs), and bispecific dual-variable- domain (DVD) antibodies or diabodies (12, 13), pose additional challenges in curation of receptor information.

**Figure 4:**
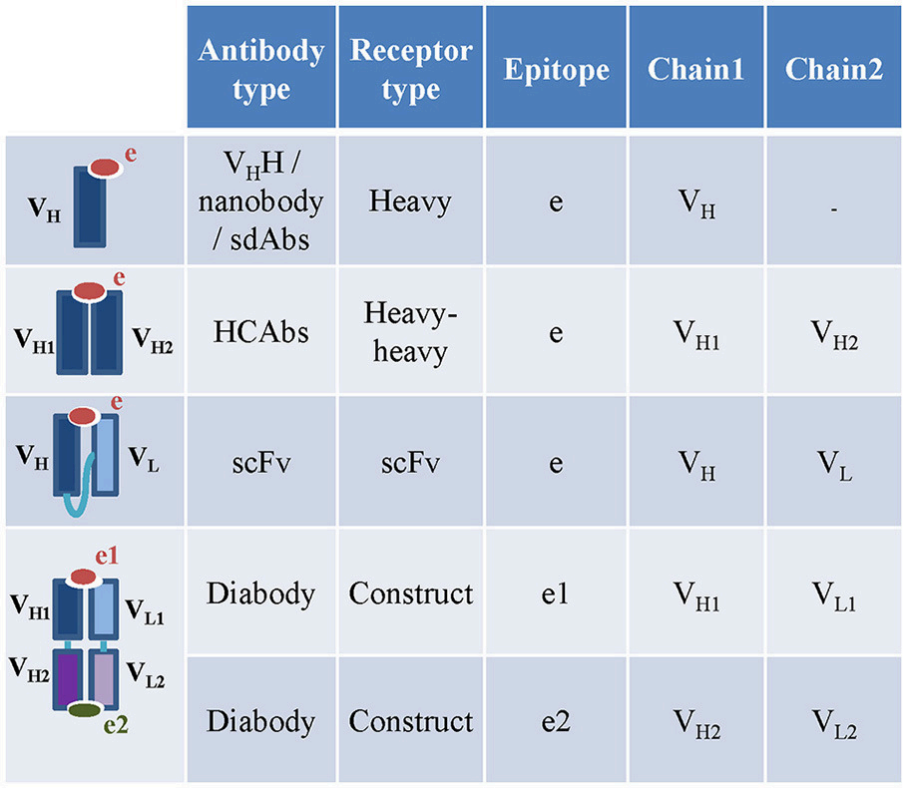
Capturing engineered, camelid and other special receptor types in the IEDB. The nanobodies and HCAbs in the IEDB are captures under heavy and heavy-heavy receptor types. The heavy and light chain variable domains in the scFv are captured as individual chains under scFv receptor type. The diabodies are captured as constructs. The heavy and light chain pairs in the diabodies which bind to two different epitopes are captured as two different assays.

To date, the available camelid and shark HCAbs curated in the IEDB-3D were engineered single-variable-domain antibodies (monomeric nanobodies or vNAR), so these were captured under receptor type “heavy” (Figure 4). ANARCI software cannot assign variable domain sequences and CDRs to IgNARs, so we captured IgNARs by manual curation, but were not able to assign calculated CDRs, gene usage and variable domains to these receptors. The sdAbs are either heavy or light chain variable domain antibodies (13). Therefore, they were captured as receptor type “heavy” or “light.” Engineered single chain antibodies (scFv) and single chain TCRs (TscFv) with full length sequences were split into their individual variable domains (heavy, light, α or β) before populating the assay table (Figure 4). The receptor type “construct” is included to capture additional types of engineered antibodies and TCRs, e.g., engineered bi-specific diabodies. The diabodies or dual-variable-domain (DVD) antibodies with two pairs of variable heavy and light domains were also split into individual pair of heavy and light variable domains. Only the author specified pair of heavy and light variable domains in the diabodies that interacts with the epitope were stored in the assay table. If the 3D structure of a diabody bound to a single epitope was solved by authors, then the pair of heavy and light chain variable domains interacting with the antigen was identified using the IEDB-calculated receptor-antigen contacts within 4Å atomic distance. If both pairs of heavy and light chain variable domains were in contact with two different antigens, then they were stored as two different receptors.

### Re-curation

The process of extending the IEDB database and reviewing previously captured data resulted in the identification and correction of curation errors, as well as merging of duplicate records. We identified cases where the chain sequences were missing from the 3D data, as well as cases where the chain type was incorrect. The Ig domains from the 3D assays were identified based on chain lengths and presence or absence of the binding chain using an in-house script. The CDR sequences and their positions were extracted using another in-house script utilizing outputs from an ANARCI (9) analysis that assigns IMGT numbering to the receptor chain sequences, and identifies the chain types (heavy, light, α, and β). Conflicts between calculated and curated Ig domains and chain types were resolved by manual re-curation of the articles. We also identified a few TCR and MHC assays where MHC allele names did not follow the correct nomenclature or were insufficiently specified. Such alleles were re-curated using an in-house script to identify the MHC allele based on their epitope binding groove domains [or G-domain (14)] sequence identity to known MHC alleles captured in the MRO database (15). G-domains are composed of α1 and α2 domains in MHC class I molecules and α1 and β1 domains in MHC class II molecules, and were identified from MHC chain sequences using IMGT MHC G-domain numbering (14). These changes in MHC allele names were verified using manual re-curation.

### Identifying data for curation

To date, we have identified 1,604 references having TCR or antibody sequence information from several strategies. One ongoing strategy is the introduction of screening all newly published articles relevant to the IEDB scope for receptor sequence information during our regular manual screen step (16). This process was introduced into our normal workflow, which includes an automated PubMed query (17) that is run every 2 weeks followed by an automatic document classifier that excludes articles highly likely to not have any epitope specific information, and manually reviewing the remaining articles. We also sought out public resources that capture information on antibody or TCR sequences. We searched the ATLAS (18), McPAS (19), VDJdb databases (20), and the Adaptive Biotechnologies website for references to journal articles that contain epitope specific receptor information and downloaded all PubMed IDs. These identified articles were manually reviewed to ascertain if the receptors mentioned were epitope specific. If an article contained such data, we manually curated the entire article following the established IEDB curation rules (16). We also screened publications with links to GenBank entries to determine if the entry is an adaptive immune receptor utilizing ANARCI to identify TCR and antibody protein sequences. We then manually screened the associated publications and curated them when they were found to contain epitope specific data. We have curated 22,510 of these for antibody or TCR sequence data and are continuing to curate the remainder on an ongoing basis. We also added TCR sequence information to articles having TCR transgenic mice as the host, wherever clear TCR sequences were available for these mice. All previously curated assays having 3D structures were reviewed and receptor sequence data were verified for accuracy and gene usage, V domains, and CDR3 sequences were calculated. These calculations have been implemented as an ongoing automated process for all newly curated 3D structures.

## Querying IEDB for epitope specific antibodies and TCRs

### Addition of receptor specific query interface

**Figure 5:**
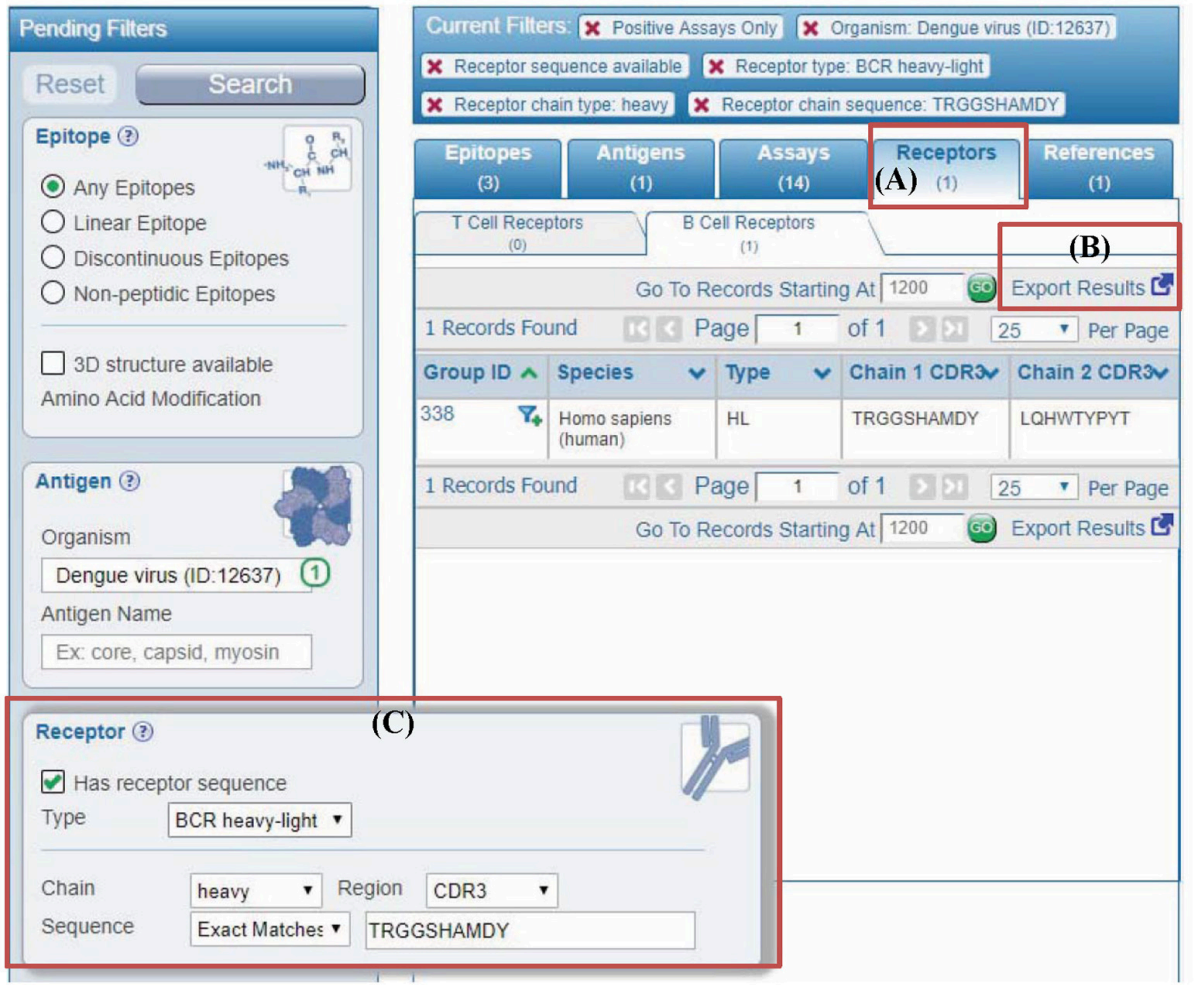
Querying IEDB using antibody or TCR sequences. **(A)** In the past, user query results in the IEDB were displayed in different tabs named epitopes, antigens, assays, and references. We added a new results tab for receptors to display different receptor groups corresponding to the user query. **(B)** All the receptor information in results can be downloaded using “export results” link in the “receptors” tab. Similarly, more detailed results are downloaded from “assays” tab. **(C)** Users can filter any query results by receptor full length protein or CDR sequences using the new receptor search panel. The example shown is to filter results by antibody (receptor type is BCR heavy-light) heavy chain with “CSYAGGKSLV” as CDR3 sequence.

To enable queries for receptor data in the IEDB, we added a new set of parameters to the “refine search results” page that is available after starting a search from the IEDB home page. Figure 5C depicts the parameters that are available, which include limiting results to those where any receptor information is available, and more specifically querying for receptor type, such as for α-β chain TCR data or heavy-light chain antibodies. Moreover, users can search by a CDR sequence or a full length receptor protein sequence with the added feature of searching for exact identity or for matches at 60, 70, 80, or 90% identity, as well as a substring match (Figure 5). Importantly, any such queries can be combined with the general IEDB search criteria, such as limiting the results to receptors recognizing viruses, or those present on T cells producing IL-10 upon epitope recognition.

### Report of receptor groups matching any IEDB query

The receptors groups matching any query in the IEDB are displayed in the newly added “receptor” tab (Figure 5A). This receptor tab describes receptor group IDs, receptor types, and their host organisms along with CDR3 sequences. All information on the receptors pertaining to the query can be downloaded in the CSV format from “export results” link on “receptor” tab (Figure 5B). Similarly, detailed query results including information on assay, immunization, epitopes, and receptors can be downloaded in the CSV format from “Assays” tab.

**Figure 6:**
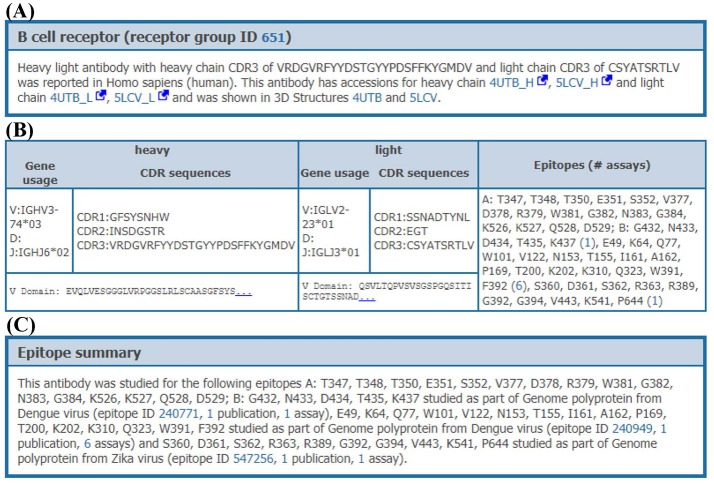
Receptor details. Receptor details are split into 3 sections. **(A)** The first section is a short summary of receptor group. This section has information on accessions of receptor chains and PDB IDs of receptor-antigen complexes involving individual receptors from this receptor group, if available. **(B)** The second section provides information of individual receptors in the receptor group. This section provides CDR sequences, VDJ gene usage, variable domain sequences and epitopes which are recognized by each receptor. **(C)** The last section provides a short summary of epitopes recognized by receptor group including assays and publications, e.g., an antibody in group ID 651 recognizes two different epitopes from Dengue and one epitope from Zika genome polyproteins.

When clicking on the receptor group ID, all data on the distinct receptors matching this group (organism, receptor type, CDR3 sequences, and variable domain sequences) are provided to the users with a comprehensive overview of the data available within the IEDB for these receptors (Figure 6). All experimental assays utilizing any given receptor can be retrieved, enabling full access to all biological activities, immunological responses and associated cellular phenotypes, binding constants, and 3D structures available for each receptor, across all epitopes that they were shown to recognize. For example, the human monoclonal antibody (receptor group ID: 651) shown in Figure 6 has been tested against two Dengue virus epitopes and one Zika virus epitope in a total of 4 neutralization assays, two ELISA qualitative binding assays and two 3D structural assays with antibody-antigen complexes (PDB IDs: 4UTB and 5LCV).

### Exports of complete receptor datasets

In addition to the targeted query described above, the entire receptor data in the IEDB can be downloaded from the “Database Export” option from “More IEDB” drop-down menu on IEDB website as a zipped CSV file (http://www.iedb.org/database_export_v3.php). This export file contains extensive details on assays, immunization, epitopes, and receptors.

## Summary of epitope specific receptor content captured so far

**Figure 7:**
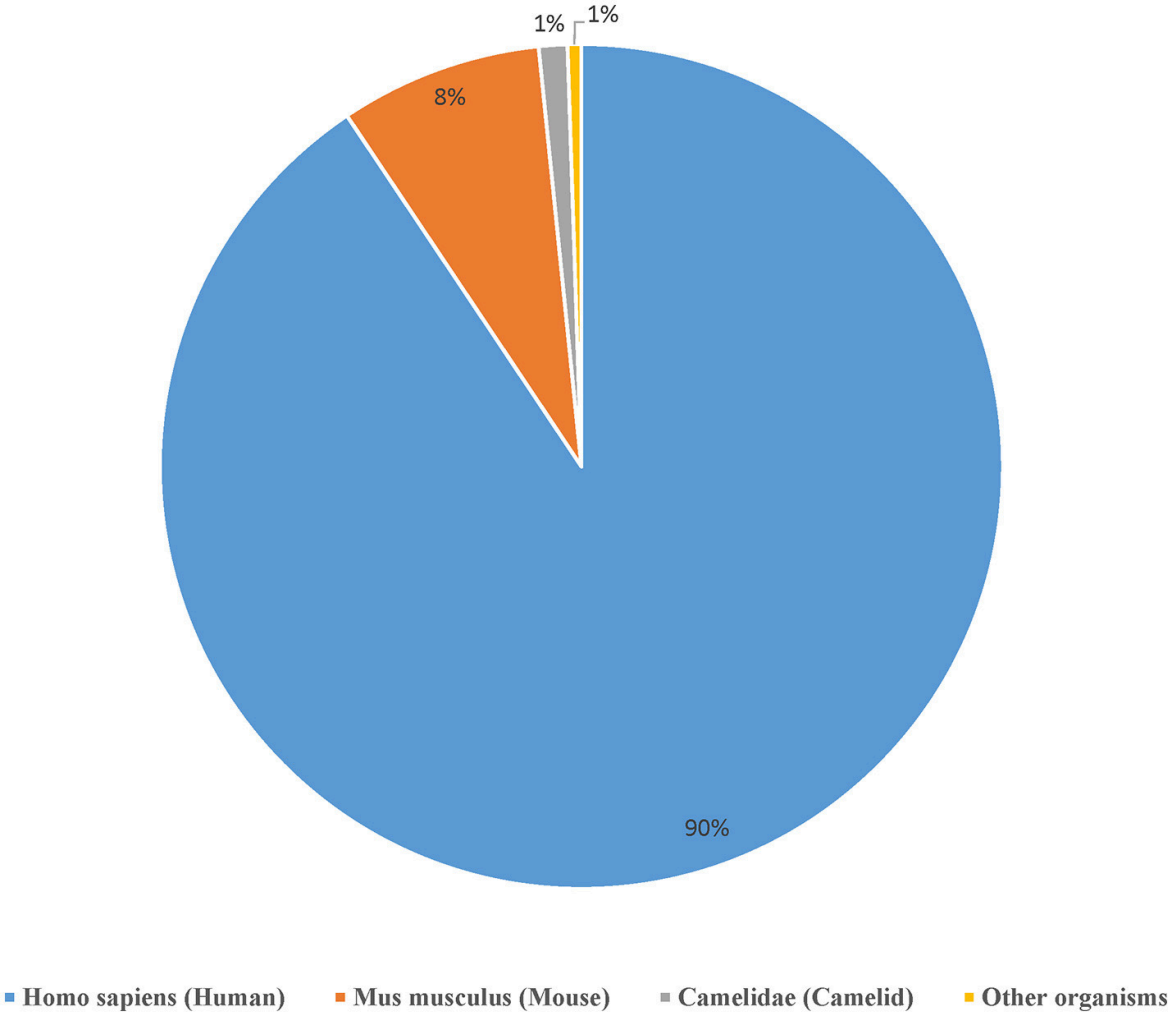
Distribution of the available receptors from different organisms. Over 90% of the antigen receptor data in the IEDB are from humans and around 8% from mice.

We curated a total of 22,510 receptors which are known to bind to 2,241 distinct epitopes in 9,901 assays from 1,604 publications as of August 2018 (Table 2). A total of 4,874 curated chains had full length protein sequences and 5,526 chains had nucleotide sequences. These 22,510 curated receptors were grouped into a total of 19,537 distinct receptors (Table 2) with 21,066 distinct chains. The distribution of distinct receptors in different organisms is shown in Figure 7. Over 90% of the distinct receptors were from humans and 8% from mice. A total of 2,319 distinct receptors had paired CDRs. All the distinct receptors were further clustered into 18,292 receptor groups, out of which 16,949 were for TCR groups and 1,343 were antibody groups.

**Table 2:** Receptor groups.

**Category**	**Number of receptors**	**Number of epitopes**
Total curated receptors	22,510	2,241
Distinct receptors	19,537	
Receptor groups	18,292	
TCR groups	16,949	536
BCR groups	1,343	1,714

## Discussion

We here report our efforts to better represent epitope specific BCR and TCR data in the IEDB. As mentioned, this is not the first such effort. Epitope-specific BCR and TCR sequences have been curated as a part of 3D structural databases such as IEDB-3D (21) and IMGT/3Dstructure-DB (22). The Epitome (23), SabDab (24), and STCRDab (25) databases store information on 3D antibody-antigen (Ab-Ag) complexes, where the focus of SabDab and STCRDab is unbound antibody and TCR structures, respectively. A complementary resource, IMGT database (2), stores germline sequences of antibodies and TCRs. Recently published databases, such as VDJdb (20) and McPAS-TCR (19), are focused on curating CDR3 sequences of TCRs from Rep-Seq experiments (6). VDJdb stores epitope specific TCR-pMHC data, while McPAS-TCR curates TCR sequences with their cognate antigens, and associated pathologies. Many of our design decisions reported here were informed by inspecting how these other databases represented immune receptors, and were aimed at creating a unifying representation of immune receptor data that is appropriate across different applications.

The IEDB is the only resource that provides information related to the host, such as species, gender age, and importantly what the host was exposed to, infected by or allergic to and other information relevant to the host's immune response, such as which cytokines are produced by T cells or if the antibodies are neutralizing and so on. With our updated curation scheme, much more information regarding BCR and TCR receptors can now easily be linked to the epitopes they bind and the immune responses associated with them in the IEDB. We have curated BCR and TCR sequence information from the past articles with low-throughput data as well as the recent articles with the high-throughput data, unlike VDJdb and McPAS-TCR databases which focus on the high-throughput data only. This task was not without its challenges. While a large amount of sequencing data has been becoming available in the literature; the vast majority of this data is not epitope specific. IEDB curators must screen all such publications related to TCR and antibody data to find the relevant records that can be curated. In many cases when receptor data is presented as being epitope specific, the epitope that it is specific for is not clearly defined. This occurs when authors sequence a large number of receptors specific to a variety of epitopes derived from the same pathogen but present CDR3 sequences in tables that do not specify which receptor was bound to which epitope.

Differences in formatting have also been a challenge as different authors describe VDJ gene usage using differing nomenclatures and describe CDR sequences using different numbering schemes especially for antibodies (26–29). Different receptor numbering schemes and the author reported CDR sequences from repertoire sequencing experiments can also include additional flanking junction region residues as a part of the CDR which create inconsistencies in storing the CDR sequences from different sources. Other related receptor sequence databases provide CDR3 sequences from TCRs with the conserved flanking anchor residues such as Cys and Phe or Cys and Trp. Such conserved anchor residues are not present for CDR1 and CDR2 sequences and also, they are excluded from the CDR regions in the IMGT numbering scheme. To provide consistent information based on the IMGT numbering scheme, we have not included the conserved anchor residues in any CDR sequences in the IEDB. We expect that as the field matures, standards for reporting experimental protocol and analysis of receptor repertoire data such as those developed by the AIRR community (30, 31) will become widely adopted, and these issues will resolve over time.

Lastly, a key challenge for the IEDB is to define what identifies a truly epitope specific receptor. The experimental procedures used to isolate and sequence receptors can be quite variable and can result in more or less stringency in what is deemed “epitope specific.” For example, one author may simply re-stimulate a PBMC culture with a peptide and sequence and report all receptors from the culture (low stringency). The use of or lack of experimental controls also varies widely, with some authors demonstrating that the epitope specific receptor is not found in controls, while others may have no such controls. We are in the process of establishing curation rules for receptor data to take these variables into account, with the goal of consistent and accurate receptor curation.

While the field is maturing, the IEDB curation procedures are adapting. This means that the exact data structure utilized might change, and the persistence of receptor identifiers cannot yet be guaranteed. We expect receptor identifiers to be stable by the end of 2018, and will at that point adhere to FAIR standards (32).

## Data availability statement

The datasets generated in this study can be found at http://www.iedb.org/database_export_v3.php.

## Author contributions

BP, SM, RV, and AS conceived and designed the work. RV, DS, and LZ contributed to the data curation. All the authors contributed to the verification of the data and development of data curation rules. SM, RV, and BP performed data analysis. SM developed the computational tools. All the authors contributed in writing and reviewing the manuscript.

### Conflict of interest statement

The authors declare that the research was conducted in the absence of any commercial or financial relationships that could be construed as a potential conflict of interest.
